# Female genitalia of *Seasogonia* Young from China, with a new synonym and a new record (Hemiptera, Cicadellidae, Cicadellini)

**DOI:** 10.3897/zookeys.164.2132

**Published:** 2012-01-11

**Authors:** Ze-hong Meng, Mao-fa Yang

**Affiliations:** 1 Institute of Entomology, Guizhou University; the Provincial Key Laboratory for Agricultural Pest Management of Mountainous Region, Guiyang, Guizhou, 550025, China

**Keywords:** Membracoidea, Auchenorrhyncha, Cicadellinae, sharpshooter, morphology, taxonomy

## Abstract

*Seasogonia* Young, 1986 is a sharpshooter genus with 13 species, four of them recorded from China. In this paper, *Seasogonia sandaracata* (Distant, 1908) is recorded as new for China and *Seasogonia rufipenna* Li & Wang, 1992 is regarded as a junior synonym of *Seasogonia nigromaculata* Kuoh, 1991. The morphological diversity of the female genitalia of *Seasogonia* is still poorly known. We provide herein detailed descriptions and illustrations of three Chinese *Seasogonia* species. Notes on the female genitalia of *Seasogonia*, including intraspecific and interspecific variation, and comparisons between the female genitalia of *Seasogonia* and of other related genera from China are provided. The preliminary results indicate that the female genitalia may provide useful features for the taxonomy of *Seasogonia* and other members of the Old World Cicadellini.

## Introduction

The sharpshooter genus *Seasogonia* was established by Young (1986) for nine species (including five new ones), with *Tettigoniella dunsiriensis* Distant, 1908 as the type species. There was no species recorded from China at that time. Kuoh (1991) described two new species of *Seasogonia* from China, and later Li and Wang (1992) described an additional one. Kuoh and Zhuo (1996) described another new species, *Seasogonia sanguinea*, which was treated as a junior synonym of *Seasogonia indosinica* (Jacobi) (Cai and Huang 1999). Wilson et al. (2009) included in the genus the species *Seasogonia sikhimensis* (Distant), which was treated as species of uncertain position by Young (1986). Until now, 13 species are known in the world and four from China. In this paper, *Seasogonia sandaracata* (Distant) is recorded as new for China and *Seasogonia rufipenna* Li & Wang is proposed as a junior synonym of *Seasogonia nigromaculata* Kuoh.

The female genitalia have yielded useful characters for the taxonomy of sharpshooters (Nielson 1965, Mejdalani 1998, Rodrigues and Mejdalani 2009, Mejdalani and Silva 2010), but the morphological diversity of the female genitalia is still poorly known compared to our current knowledge of the male genitalia. In the monograph of Young (1986), the female genitalia of *Seasogonia* species were only briefly described. The present study provides a detailed description of the previously unknown female genitalia of three Chinese *Seasogonia* species. Notes on the female genitalia of *Seasogonia*, including intraspecific and interspecific variation, and comparisons between the female genitalia of *Seasogonia* and of other related genera from China are provided. We hope that this description will point out useful characters for the taxonomic studies on the Old World Cicadellini.

## Material and methods

The male and female genital structures were prepared according to the techniques described by Oman (1949) and Mejdalani (1998), respectively. The dissected parts in glycerin were stored in microvials and the microvial was attached below the respective specimen to which the genitalia pars belonged. The morphological terminology of the female genitalia follows mainly Davis (1975) and Nielson (1965). Use of the term gonoplac (=valvula III) and the names for the processes of the dorsal and ventral sculptured areas of the first ovipositor valvula follow Mejdalani (1998). The illustrations of the ventral view of basal region of female genitalia are based on undisturbed and intact female genitalia that the valvulae are not separated. Most of specimens studied are housed in the Institute of Entomology, Guizhou University, Guiyang, China (GUGC), and some are deposited in Shanghai Entomological Museum, Chinese Academy of Sciences (SEMCAS). Abbreviations used in this paper are as follows: AP = articulation point, DE = denticles, LB = lobe, PP = preapical prominence, TO = tooth, VHA = ventral hyaline area, VID = ventral interlocking device.

## Results

### List of Chinese Seasogonia species and their female genitalia

#### Seasogonia indosinica

(Jacobi)

http://species-id.net/wiki/Seasogonia_indosinica

Figs 1–3 26 34

Tettigoniella indosinica Jacobi, 1905: 445
Seasogonia sanguinea Kuoh & Zhuo, 1996: 1

##### Material examined.

1 male, China, Guangxi Province, Huaping, 5 June 1997, coll. Yang Mao-fa; 3 males, 2 females, China, Guangxi Province, Jinxiu County, Dayaoshan, Alt. 500m, 28 April 2008, coll. Meng Ze-hong; 2 males, China, Hainan Province, Jianfengling, 14–15 May 1997, coll. Yang Mao-fa; 1 male, China, Hainan Province, Jianfengling, 17 April 2009, coll. Yang Zai-hua; 14 males, China, Hainan Province, Diaoluoshan, 10–12 April 2009, coll. Yang Zai-hua; 1 male, China, Hainan Province, Bawangling, 24 April 2009, coll. Yang Zai-hua; 1 male, China, Sichuan Province, Emeishan, 14 July 1995, coll. Yang Mao-fa; 3 males, China, Guizhou Province, Guiyang City, 5 June 1981, coll. Li Zi-zhong and Ma Gui-yan; 1 male, China, Guizhou Province, Guiyang City, 2 July 1986, coll. Li Zi-zhong; 4 males, 1 female, China, Guizhou Province, Guiyang City, 15 June 1992, coll. Zhang Yu-qiong; 10 males, China, Guizhou Province, Taijiang County, 9–17 May 1985, coll. Li Zi-zhong; 17 males, China, Guizhou Province, Libo County, 19–24 May 1995, coll. Chen Xiang-sheng; 8 males, China, Guizhou Province, Libo County, 24–30 May 1998, coll. Li Zi-zhong and Song Qiong-zhang; 2 males, China, Guizhou Province, Libo County, 14–17 June 2006, coll. Zhou Zhong-hui and Zhang Bin; 5 males, 3 females, China, Guizhou Province, Chishui County, 28 May 2000, Li Zi-zhong and Chen Xiang-sheng; 2 males, 3 females, China, Guizhou Province, Xishui City, 3 June, coll. Li Zi-zhong and Chen Xiang-sheng; 2 males, China, Guizhou Province, Chishui City, 28–30 May 2006, coll. Tang Yi and Yang Zai-hua; 1 male, China, Guizhou Province, Fanjingshan, 27 July 2001, coll. Li Zi-zhong; 2 males, 2 females, China, Guizhou Province, Fanjingshan, 2–3 June 2002, coll. Li Zi-zhong and Yang Mao-fa; 12 males, 7 females, China, Guizhou Province, Daozhen County, Dashahe, 22–27 May 2004, coll. Zhang Bin, Song Qiong-zhang, Xu Fang-ling and Chen Xiang-sheng; 39 males, 4 females, China, Guizhou Province, Leigongshan, 31 May to 5 June 2005, coll. Tang Yi, Li Zi-zhong, Zhang Bin, Song Qiong-zhang, Zhang Zheng-guang, Ge De-yan, Yang Zai-hua and Xu Fang-ling; 1 male, China, Guizhou Province, Anshun City, 20 July 2005, coll. Zhou Zhong-hui; 3 males, China, Guizhou Province, Duyun City, 5 May 2006, coll. Yang Zai-hua and Zhou Zhong-hui; 4 males, China, Guizhou Province, ShiBing County, Yuntaishan, 20–21 May 2009, coll. Yang Zai-hua; 43 males, 10 females, China, Guizhou Province, Suiyang County, Kuankuoshui, 2–9 June 2010, coll. Dai Ren-huai, Song Qiong-zhang, Li Hu, Li Yu-jian, Zhang Bin, Zheng Yan-li and Xing Ji-chun.

##### Distribution.

Myanmar, Vietnam, India, China (Fujian, Guangxi, Hainan, Sichuan, Guizhou, Yunnan).

##### Female genitalia.

Abdominal sternite VII (Fig. 26), in ventral view, broader than long; anterior margin straight; posterior margin well produced medially, sometimes forming two distinct lateral lobes; lateral margins convergent posteriorly; surface with few small setae mostly on basal half. Internal sternite VIII not forming sclerites. Pygofer (Fig. 27), in lateral view, slightly produced posteriorly; posterior margin with subacute apex; surface with macrosetae on posterior portion and extending anteriorly along ventral margin, attaining midlength. Valvifers I, in lateral view (Fig. 28), nearly rectangular, slightly expanded posteriorly, posteroventral margin angulate; in ventral view (Fig. 34), forming lobes (LB) articulating with valvulae I. Valvifers II (Fig. 29), in lateral view, nearly fusiform, with small group of clustered setae near articulation point (AP). Valvulae I of ovipositor, in ventral view (Fig. 34), with base gradually broadening posteriorly; in lateral view (Fig. 30), shaft distinctly curved dorsally and with dorsal margin concave, with ventral hyaline area (VHA) near apex; dorsal sculptured area located on apical half, broadening to near apex and gradually narrowing to apex, formed by dense scale-like processes; ventral sculptured area restricted to apical portion, formed by dense imbricate processes; ventral interlocking device (VID) distinct on basal 2/3 of shaft; apex of shaft acute. Valvulae II of ovipositor (Figs 31 and 32), in lateral view, slightly expanded beyond basal curvature, distinctly curved dorsally and with dorsal margin concave; apex narrowly rounded; preapical prominence (PP) indistinct; shaft bearing approximately 4–6 teeth (TO) distributed on apical half behind basal curvature; each tooth semiround and bearing few denticles (DE) or not bearing denticles; denticles mostly distributed on dorsal margin of shaft between teeth and on dorsal and ventral margins of apical portion, dentate dorsal margin longer than ventral margin; ducts extending toward dorsal margin and toward apical portion of shaft. Gonoplacs (Fig. 33), in lateral view, with basal half narrow and apical half distinctly expanded; apex rounded.

**Figures 1–12.**
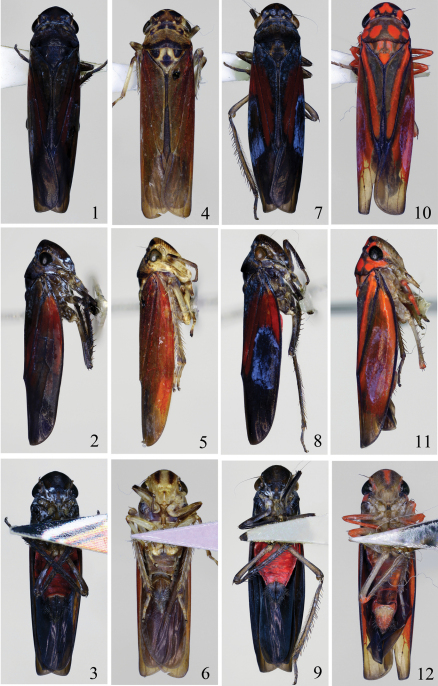
*Seasogonia indosinica* (Jacobi), body of male (9.0 mm): **1** dorsal view **2** lateral view **3** ventral view. *Seasogonia nigromaculata* Kuoh, body of male (11.5 mm): **4** dorsal view **5** lateral view **6** ventral view. *Seasogonia rosea* Kuoh, body of male (10.9 mm): **7** dorsal view **8** lateral view **9** ventral view. *Seasogonia sandaracata* (Distant), body of male (11.2 mm): **10** dorsal view **11** lateral view **12** ventral view.

#### Seasogonia nigromaculata

Kuoh

http://species-id.net/wiki/Seasogonia_nigromaculata

Figs 4–6 13–19

Seasogonia nigromaculata Kuoh, 1991: 165
Seasogonia rufipenna Li & Wang, 1992: 98,syn. n.

##### Notes.

We checked the holotype of *Seasogonia rufipenna* Li & Wang. Unfortunately, we failed to check the holotype of *Seasogonia nigromaculata* Kuoh, but based on its detailed Chinese descriptions and illustrations of external feature and male genitalia provided by Kuoh (1991), we regarded *Seasogonia rufipenna* Li & Wang as a junior synonym of *Seasogonia nigromaculata* Kuoh.

##### Material examined.

Male,holotype of *Seasogonia rufipenna*, Guizhou Province, Taijiang County, Shihuihe, 16 May 1985, coll. Li Zi-zhong.

##### Distribution.

China (Guizhou, Yunnan).

##### Male genitalia.

**Figures 13–25.**
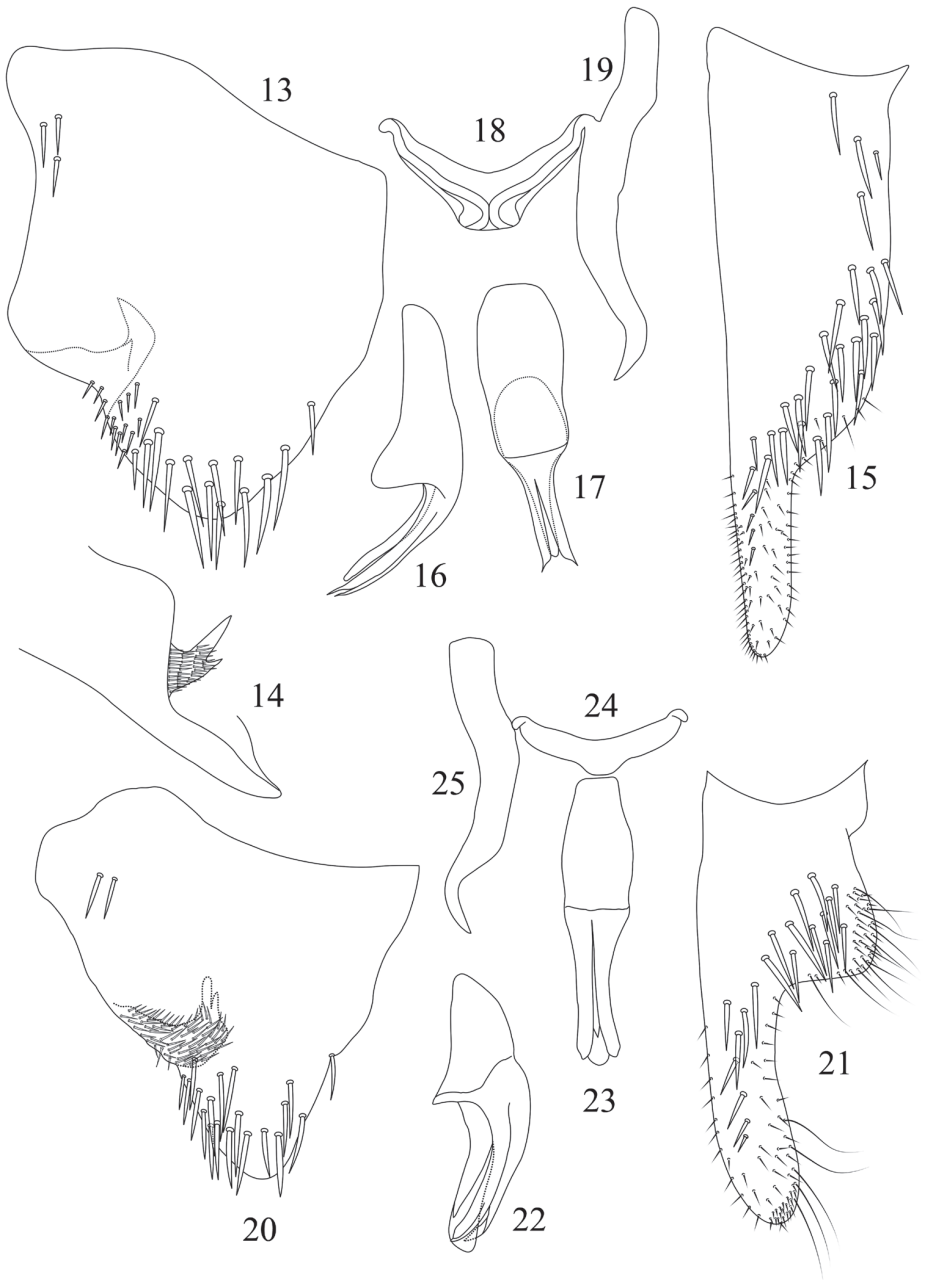
*Seasogonia nigromaculata* Kuoh, male genitalia: **13** pygofer, lateral view **14** pygofer process, caudal view **15** subgenital plate, ventral view **16** aedeagus, lateral view **17** aedeagus, ventral view **18** connective, dorsal view **19** style, dorsal view. *Seasogonia rosea* Kuoh, male genitalia: **20** pygofer, lateral view **21** subgenital plate, ventral view **22** aedeagus, lateral view **23** aedeagus, ventral view **24** connective, dorsal view **25** style, dorsal view.

Pygofer in lateral view (Fig. 13), with broad base and gradually narrowed posteriorly, posterodorsal margin concave; apex narrowly rounded; with two or three macrosetae on basiventral portion and many macrosetae on posterior portion; pygofer process (Figs 13 and 14) arising near median-ventral margin, extending dorsally, bifurcate apically and divided into two processes and with small process as 1/3 long as the other one; with dense setae on basal and median portion. Subgenital plate (Fig. 15) with multiseriate macrosetae on broad basal two-third portion, with uniseriate macrosetae and some short microsetae on narrowed apical one-third portion. Aedeagus (Figs 16 and 17) broad at basal half and with subrounded median-dorsal process; shaft slender and with paired ventral processes diverging from base of shaft, processes with apex acute and exceeding apex of shaft. Connective (Fig. 18) Y-shaped, stalk short. Style (Fig. 19) slightly unciform apically.

##### Female genitalia.

Unknown.

**Figures 26–33.**
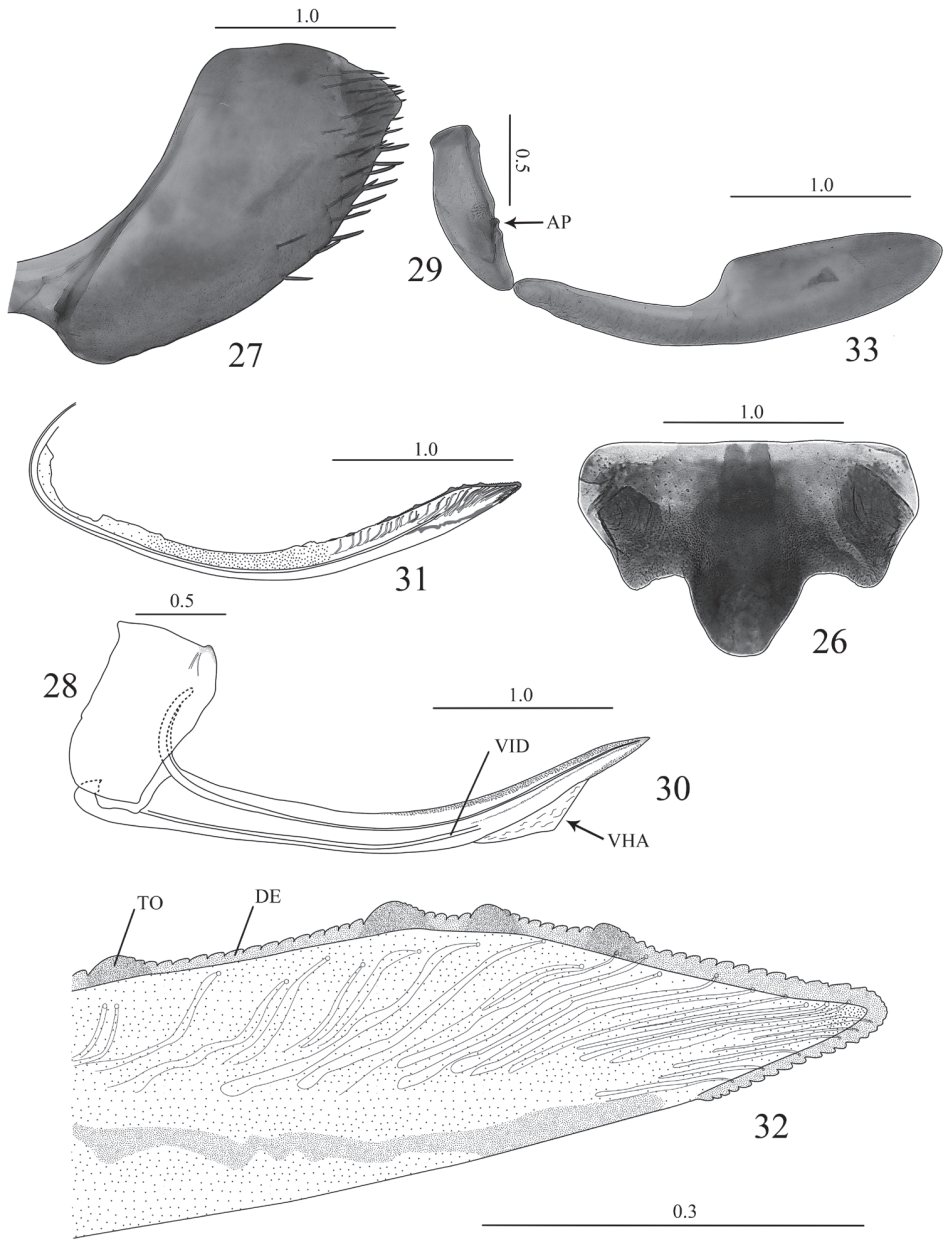
*Seasogonia indosinica* (Jacobi), female genitalia: **26** sternite VII, ventral view **27** Pygofer, lateral view **28** valvifer I, lateral view **29** valvifer II, lateral view **30** valvula I, lateral view **31** valvula II, lateral view **32** apex of valvula II, lateral view **33** gonoplac, lateral view. AP = articulation point, DE = denticles, TO = tooth, VHA = ventral hyaline area, VID = ventral interlocking device. Scale bars in millimeters.

#### Seasogonia rosea

Kuoh

http://species-id.net/wiki/Seasogonia_rosea

Figs 7–9 20–25 35–37

Seasogonia rosea Kuoh, 1991: 166

##### Material examined.

1 female, China, Yunnan Province, Tengchong County, Shangyun Village, Alt. 1700–1900m, 15 July 2002, coll. Yang Mao-fa; 1 male, 1 female, China, Yunnan Province, Tengchong County, Gaoligongshan, Alt. 1900–2000m, 17 July 2002, coll. Yang Mao-fa and Song Hong-yan; 21 males, 7 females, China, Yunnan Province, Tengchong County, Gaoligongshan, Baihualing, Alt. 1800–2400m, 28 May to 3 June 2009, coll. Yang Zai-hua and Li Bin; 10 males, 9 females, China, Yunnan Province, Tengchong County, Gaoligongshan, 13–15 June 2011, coll. Yang Zai-hua and Li Yu-jian; 1 male, 1 female, China, Yunnan Province, Yingjiang County, Tongbiguan, Alt. 1400–1500m, 20 July 2002, coll. Yang Mao-fa and Song Hong-yan; 19 males, China, Yunnan Province, Yingjiang County, Tongbiguan, 1–3 June 2011, coll. Yang Zai-hua and Li Yu-jian; 7 males, China, Yunnan Province, Fugong County, Shangpa Town, 17–18 May 2010, coll. Ni Jun-qiang, Li Hu and Zhang Pei.

##### Distribution.

China (Yunnan).

##### Male genitalia.

Pygofer (Fig. 20) in lateral view, broad and triangular, gradually narrowed posteriorly; with several macrosetae on basiventral portion and many macrosetae on posterior portion; pygofer process arising near median-ventral margin, extending dorsally, bifurcate apically and divided into two processes, short process acute and nearly as 2/3 long as the other one; surface with dense setae except apex. Subgenital plate (Fig. 21) with anterior half broad, surface with multiseriate macrosetae on basal one-half and with uniseriate macrosetae and some short microsetae on posterior half. Aedeagus (Figs 22 and 23) broad at basal half and with angulate median-dorsal process; shaft with acute internal process and with paired ventral processes diverging from base of shaft, ventral processes with apex acute and extending as long as apex of shaft. Connective (Fig. 24) broad, V-shaped. Style (Fig. 25) slightly unciform apically.

##### Female genitalia.

Abdominal sternite VII (Fig. 35), in ventral view, with posterior margin conspicuously more produced medially than in *Seasogonia indosinica*. Other characteristics as in *Seasogonia indosinica*.

**Figures 34–40.**
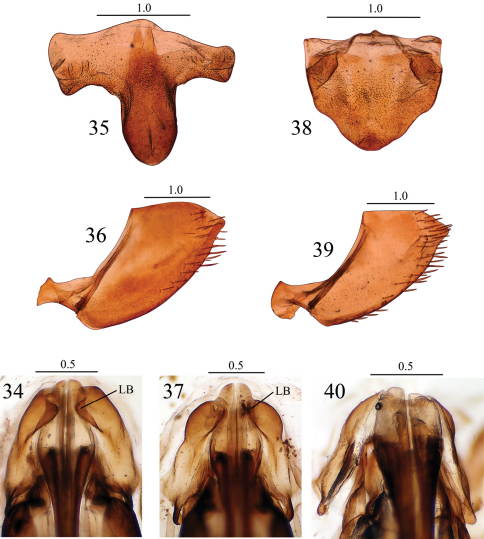
*Seasogonia indosinica* (Jacobi): **34** valvifers I and bases of valvulae I, ventral view *Seasogonia rosea* Kuoh: **35** Sternite VII, ventral view **36** pygofer, lateral view **37** valvifers I and bases of valvulae I, ventral view *Seasogonia sandaracata* (Distant): **38** Sternite VII, ventral view **39** pygofer, lateral view **40** valvifers I and bases of valvulae I, ventral view. LB = lobe. Scale bars in millimeters.

#### Seasogonia sandaracata

(Distant)

http://species-id.net/wiki/Seasogonia_sandaracata

Figs 10–12 38–40 41–42 53–54

Tettigoniella sandaracata Distant, 1908: 217

##### Material examined.

5 males, 1 female, China, Yunnan Province, Yingjiang County, Tongbiguan, Alt. 1200m, 15 June 2001, coll. Tian Ming-yi; 10 females, China, Yunnan Province, Yingjiang County, Tongbiguan, Alt. 1400–1500m, 20 July 2002, coll. Yang Mao-fa, Li Zi-zhong, Song Hong-yan and Dai Ren-huai; 32males, 7 females, China, Yunnan Province, Yingjiang County, 29 May to 3 June 2011, coll. Yang Zai-hua and Li Yu-jian; 13 males, 2 females, China, Yunnan Province, Yingjiang County, Xima Town, Alt. 1700m, 8–10 June 2009, coll. Yang Zai-hua and Li Bin; 2 males, 1 female, China, Yunnan Province, Yingjiang County, Tongbiguan, Alt. 270m, 13 June 2009, coll. Yang Zai-hua and Li Bin; 1 male, 11 females, China, Yunnan Province, Tengchong County, Shangyun Village, Alt. 1400m, 14 July 2002, coll. Li Zi-zhong and Yang Mao-fa; 1 male, 3 females, China, Yunnan Province, Tengchong County, Gaoligongshan, Alt. 1900–2000m, 17 July 2002, coll. Yang Mao-fa, Li Zi-zhong, and Song Hong-yan; 5 females, China, Yunnan Province, Longling County, Longxin, Alt. 1800m, 24 July 2002, coll. Yang Mao-fa, Li Zi-zhong, and Song Hong-yan; 1 male, 1 female, China, Yunnan Province, Tengchong County, Gaoligongshan, Alt. 1800–2400m, 28 May to 5 June 2009, coll. Yang Zai-hua and Li Bin; 3 males, China, Yunnan Province, Ruili County, Moli, Alt. 770m, 15 June 2009, coll. Yang Zai-hua and Li Bin; 7 males, 5 females, China, Yunnan Province, Ruili County, 5–7 June 2011, coll. Li Yu-jian and Yang Zai-hua; 1 male, China, Yunnan Province, Pianma, 10 May 2010, coll. Zhang Bin; 18 males, 4 females, China, Yunnan Province, Pianma, 17–19 June 2011, coll. Li Yu-jian and Yang Zai-hua; 47 males, 30 females, China, Xizang Province, Muotuo County, 6 May to 4 June 1980, coll. Jin Gen-tao and Wu Jian-yi (Specimens are deposited in Shanghai Entomological Museum, Chinese Academy of Sciences (SEMCAS)).

##### Distribution.

India, Myanmar, China (Yunnan, Xizang). New Record for China.

##### Female genitalia.

Abdominal sternite VII (Fig. 38), in ventral view, nearly as broad as long; anterior margin straight; posterior margin broadly convex. Pygofer (Fig. 39), in lateral view, slightly produced; posterior margin with subacute apex; surface with macrosetae on posterior portion and extending anteriorly along nearly whole of ventral margin. Valvifers I, in ventral view (Fig. 40), not forming lobes articulating with valvulae I. Valvulae I, in ventral view (Fig. 40), much broader than in *Seasogonia indosinica*. Other characteristics as in *Seasogonia indosinica*.

**Figures 41–52.**
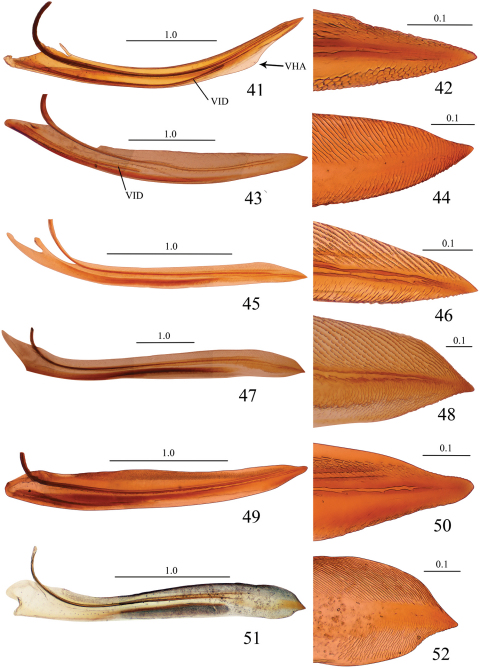
Valvulae I and their apical portions, lateral view. **41, 42** *Seasogonia sandaracata* (Distant, 1908) **43, 44** *Gununga yoshimotoi* Young, 1986 **45, 46** *Anagonalia melichari* (Distant, 1908) **47, 48** *Sphinctogonia lacta* Zhang & Kuoh, 1993 **49, 50** *Cicadella viridis* (Linnaeus, 1758) **51, 52** *Stenatkina albopennis* Yang, 2007. VHA = ventral hyaline area, VID = ventral interlocking device. Scale bars in millimeters.

**Figures 53–64.**
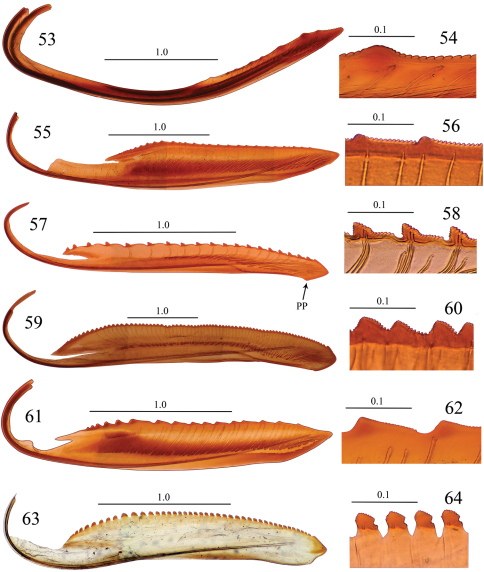
Valvulae II and their teeth, lateral view. **53, 54** *Seasogonia sandaracata* (Distant, 1908) **55, 56** *Gununga yoshimotoi* Young, 1986 **57, 58** *Anagonalia melichari* (Distant, 1908) **59, 60** *Sphinctogonia lacta* Zhang & Kuoh, 1993 **61, 62** *Cicadella viridis* (Linnaeus, 1758) **63, 64** *Stenatkina albopennis* Yang, 2007. PP = preapical prominence. Scale bars in millimeters.

### Notes on the female genitalia of Seasogonia

**Intraspecific variation.** The number of teeth on valvulae II often varied from 4–7 teeth. In addition, the location of each tooth varied among different specimens, or between each valvula of a single specimen.

**Interspecific variation.** Females of Chinese *Seasogonia* species can be distinguished from each other mainly by the following characters: (1) the posterior margin of sternite VII is well produced medially and forms a median lobe in *Seasogonia indosinica* (Fig. 26) and *Seasogonia rosea* (Fig. 35), and the latter species has the projection conspicuously more elongated; in *Seasogonia sandaracata* (Fig. 38), the sternite VII is broadly convex and lacks a distinct median lobe; (2) in *Seasogonia sandaracata*, the macrosetae on the pygofer surface extend anteriorly distinctly farther than in the other two species (Fig. 39); (3) the valvifers I, in ventral view, form lobes (LB) articulating with valvulae I in *Seasogonia indosinica* and *Seasogonia rosea* (Figs 34 and 37), and the former species has the lobes slightly larger; *Seasogonia sandaracata* lacks the lobes (Fig. 40); (4) in *Seasogonia indosinica* and *Seasogonia rosea* (Figs 34 and 37), the bases of valvulae I, in ventral view, are more slender than in *Seasogonia sandaracata* (Fig. 40). Other characteristics are little changed.

The female abdominal sternite VII overlaps the bases of the ovipositor and usually has much interspecific variation. Young (1986) stated that the female sternite VII varied from slightly to strongly convex apically. We provide the descriptions and illustrations of sternite VII of Chinese *Seasogonia* species, and the characters such as lateral and median lobes of posterior margin can efficiently distinguish Chinese *Seasogonia* species. The female abdominal sternite VII is widely used for separating species of a genus or genera in sharpshooters or several groups of other leafhoppers (Greene 1971, Young 1977, 1986, Krishnankutty and Viraktamath 2008).

The ventral view of basal region of female genitalia has been used to discriminate species in a genus of leafhoppers by some other workers (Greene 1971, Leal et al. 2009). Greene (1971) employed the characters of base of the valvulae I for distinguishing species of deltocephaline genus *Psammotettix*. Based on the present studies, as stated by Greene, the female genitalia characters did not delimit species as precisely as do the aedeagus characters of the male, but the female genitalia such as ventral view of basal region of valvulae I can be used in combination with edge of abdominal sternite VII or other female characters to separate species. Leal et al. (2009) also provided the ventral view of basal portion of female genitalia in sharpshooter genus *Scoposcartula* and discovered the new characters on base of the valvulae which named prevalvifer area and the sclerotised wall. The present study revealed the occurrence of peculiar lobes articulating with valvulae I, which can be added to the combination of features that distinguish species in the genus.

**Comparative notes on *Seasogonia* and other related genera from China.** In the present paper, the previously unknown female genitalia of three species of *Seasogonia* from China were described and illustrated for the first time. *Seasogonia* is apparently closely related to the genus *Sochinsogonia* Young, 1986 in appearance, but in *Sochinsogonia* the posterior margin of the sternite VII is concave, whereas it is convex in *Seasogonia*; in addition, the valvulae I and valvulae II are distinctly curved dorsally in *Seasogonia* and not so in *Sochinsogonia* (Young 1986).

Unfortunately, we did not have at hand specimens of *Sochinsogonia*. Thus, we compared the female genitalia of *Seasogonia* with those of some other related genera from China (Figs 41–64). Based on the female genitalia, *Seasogonia* can be distinguished from other Old World Cicadellini by the following combination of characters: (1) the posterior margin of the sternite VII is distinctly convex (26, 35 and 38); (2) the valvulae I and II are distinctly curved dorsally (Figs 30, 31, 41 and 53); (3) the dorsal sculptured area of valvulae I and the teeth of valvulae II are distributed only on the apical half of shaft; the valvulae I have a ventral hyaline area (VHA) near apex (Figs 30 and 41); only a small number of teeth is present on valvulae II (Figs 31, 32 and 53); (4) the dorsal and ventral sculptured areas of valvulae I are formed by dense scale-like processes that are not arranged in oblique lines (Figs 30, 41 and 42). It is important to mention that a considerable amount of morphological diversity is observed in the valvifers I, the base of valvulae I and the shape of teeth of valvulae II (Figs 54, 56, 58, 60, 62 and 64). The structure of the valvulae II of *Seasogonia* seems very unusual for Cicadellini in general. We compared the shape and teeth with other cicadellines, but found little similarities on our studied genera of Old world Cicadellini. Young (1977) illustrated valvulae II of many New World genera. We found that the valvulae II of *Seasogonia* are somewhat similar to the New World genus *Versigonalia* (Young 1977, Mejdalani 1998). Just as *Seasogonia* species, the valvulae II are not very expanded beyond basal curvature, shaft just have poorly developed teeth in apical one-third or one-half and apex is narrowly rounded in *Versigonalia*, but the shaft of valvulae does not curve so distinctly as in *Seasogonia* and not have clear dentate apicoventral margin.

The sclerites of the genital chamber described by Young (1986) in females of various Cicadellini are the reduced internal sternite VIII. The sclerotized parts of this sternite can provide shape-related characteristics useful for species distinctions in the subfamily (Nielson 1965, Mejdalani 1998, Takiya and Mejdalani 2004, Leal et al. 2009). We did not observe well sclerotized parts of the sternite VIII in the species of Chinese Cicadellini studied, which is consistent with Young (1986). As Mejdalani (1998) indicated, the features of the female genitalia, especially from the valvulae I and II, such as the shaft form and the teeth form and distribution, can be potentially useful taxonomic characters for the sharpshooter taxonomy. The valvulae I and II usually vary in shape, proportion, armature or texture (Dietrich 2005). We compared the valvulae I and II of some Old World species (Figs 41–64) and concluded that the features of the female genitalia were reliable and useful. The valvulae I can have a straight (Figs 43 and 49), distinctly concave (Fig. 41), or convex or angled to different degrees (Figs 47 and 51) dorsal margin. The dorsal and ventral sculptured areas of valvulae I are arranged in oblique lines (Figs 44, 46, 48 and 52) or not (Figs 42 and 50). The ventral interlocking device (VID) can extend along the basal 2/3 (Fig. 41) or 1/3 (Fig. 43) of the blade, among other proportions. The apex of valvulae I can be curved ventrally (Figs 48 and 52), dorsally (Fig. 44), or be not curved (Fig. 42). The shaft of valvulae II shows also much diversity. Its dorsal margin can be concave (Fig. 53), straight (Figs 55 and 61), or convex (Figs 59 and 63) to different degrees. The valvulae II may bear a ventral preapical prominence (Fig. 57) or not (Fig. 61). The distribution of teeth on valvulae II can be continuous (Fig. 60) or not (Fig. 64). The form of the teeth can be semiround (Fig. 54), triangular with subequal straight sides (Fig. 60), triangular with longer posterior side (Figs 56 and 62), triangular with flat posterior area (Fig. 58), or of irregular shape (Fig. 64). The preliminary results herein discussed indicate that the female genitalia can provide useful features for the taxonomy of *Seasogonia* and other members of the Old World Cicadellini.

## Supplementary Material

XML Treatment for Seasogonia indosinica

XML Treatment for Seasogonia nigromaculata

XML Treatment for Seasogonia rosea

XML Treatment for Seasogonia sandaracata
